# Multipotent Caudal Neural Progenitors Derived from Human Pluripotent Stem Cells That Give Rise to Lineages of the Central and Peripheral Nervous System

**DOI:** 10.1002/stem.1991

**Published:** 2015-05-21

**Authors:** Mark Denham, Kouichi Hasegawa, Trevelyan Menheniott, Ben Rollo, Dongcheng Zhang, Shelley Hough, Abdullah Alshawaf, Fabia Febbraro, Samiramis Ighaniyan, Jessie Leung, David A. Elliott, Donald F. Newgreen, Martin F. Pera, Mirella Dottori

**Affiliations:** ^1^ Department of Anatomy and NeurosciencesUniversity of MelbourneMelbourneAustralia; ^2^ Danish Research Institute of Translational NeuroscienceAarhus UniversityAarhusDenmark; ^3^ Institute for Integrated Cell‐Material SciencesKyoto UniversityKyotoJapan; ^4^ InStemNCBSBangaloreKarnatakaIndia; ^5^ Murdoch Children's Research InstituteMelbourneAustralia; ^6^ Centre for Neural Engineering, Department of Electrical and Electronic EngineeringUniversity of MelbourneMelbourneAustralia; ^7^ Walter and Eliza Hall InstituteMelbourneAustralia; ^8^ Florey Institute of Neuroscience and Mental HealthMelbourneAustralia

**Keywords:** Roof plate, Floor plate, Neural crest, Peripheral nervous system, Human embryonic stem cells

## Abstract

The caudal neural plate is a distinct region of the embryo that gives rise to major progenitor lineages of the developing central and peripheral nervous system, including neural crest and floor plate cells. We show that dual inhibition of the glycogen synthase kinase 3β and activin/nodal pathways by small molecules differentiate human pluripotent stem cells (hPSCs) directly into a preneuroepithelial progenitor population we named “caudal neural progenitors” (CNPs). CNPs coexpress caudal neural plate and mesoderm markers, and, share high similarities to embryonic caudal neural plate cells in their lineage differentiation potential. Exposure of CNPs to BMP2/4, sonic hedgehog, or FGF2 signaling efficiently directs their fate to neural crest/roof plate cells, floor plate cells, and caudally specified neuroepithelial cells, respectively. Neural crest derived from CNPs differentiated to neural crest derivatives and demonstrated extensive migratory properties in vivo. Importantly, we also determined the key extrinsic factors specifying CNPs from human embryonic stem cell include FGF8, canonical WNT, and IGF1. Our studies are the first to identify a multipotent neural progenitor derived from hPSCs, that is the precursor for major neural lineages of the embryonic caudal neural tube. Stem Cells *2015;33:1759–1770*

## Introduction

In the embryo, specification of early neural progenitors begins soon after gastrulation with the formation of the neural plate, which subsequently folds to become the embryonic neural tube 1, 2. The embryonic neural plate can be divided into two developmentally distinct regions, the rostral (anterior) and caudal (posterior) neural plates. Specification of the rostral neural plate occurs during the earliest stages of neural induction and gives rises to the rostral forebrain, which corresponds to the telencephalon and rostral diencephalon. The caudal neural plate gives rise to the caudal diencephalon, mesencephalon, and metencephalon and the spinal cord. Lineages associated with the caudal neural plate include ventral floor plate cells, dorsal roof plate cells, and neural crest.

Formation of the caudal neural plate is mediated by caudalizing signals, such as Wnts, retinoic acid, and FGF, secreted from the primitive streak and caudal mesoderm 1, 3. Recent studies have demonstrated that the caudal neural plate region arises from bipotent mesoectodermal progenitors, also known as axial stem cells, which coexpress mesodermal and neural markers including Sox2 and Brachyury 4, 5. Axial stem cells can generate either neural or mesodermal lineages, their fate being regulated by persistent expression of Sox2 or onset of Tbx6 expression, respectively 4, 6.

The earliest neural marker associated with the human neural plate has been demonstrated to be PAX6 7 and the derivation of PAX6 neural progenitor from human pluripotent cultures reveals a rostral identity which can be achieved by dual SMAD inhibition 8. Exposing these rostral neural plate progenitors to caudalization cues has previously been reported to shift these neural progenitors into a caudal identity 9. However, to date, the direct derivation of an early caudal neural progenitor that can generate multiple caudal neural plate derivatives has not been demonstrated.

Our previous studies described the derivation of an early neural progenitor cells derived from human pluripotent stem cell (hPSC), phenotypically defined as OCT4−/SOX2+/PAX6− 10. These progenitors are generated by 4 days treatment of hPSC with small molecule inhibitors of glycogen synthase kinase 3β (GSK3β) and Activin/Nodal signaling, called CHIR99021 (CHIR) and SB431542 (SB), respectively. Subsequent removal of the dual inhibitors results in the upregulation of the neuroepithelial marker, PAX6 10. Our studies also showed that activating the sonic hedgehog (SHH) pathway in these OCT4−/SOX2+/PAX6− progenitors results in their efficient differentiation to FOXA2+ floor plate cells, a major derivative of the caudal neural plate 10. Here, we further characterize the OCT4−/SOX2+/PAX6− progenitors and demonstrate their coexpression of mesodermal and caudal neural markers, and biased differentiation toward neural lineages of the caudal neural plate. Consistent with a caudal neural plate identity, we also show their efficient capacity to be directed to neural crest lineages. Given their phenotypic characteristics, we have renamed and further defined the OCT4−/SOX2+/PAX6− progenitors as “caudal neural progenitors” (CNPs). Finally, we determined the extrinsic signaling factors that can mediate CNP induction from hPSC are FGF8, canonical WNT, and IGF1. In summary, these studies describe a novel multipotent progenitor lineage derived from hPSCs, which are the precursor cells for caudal neural plate lineages, and identify the signaling pathways involved in inducing CNP fate.

## Materials and Methods

### Human Embryonic Stem Cell Culture

HES‐3 (WiCell, Madison, WI, www.wicell.org), ENVY‐HES‐3 (BioTime, Alameda, CA, USA, www.biotimeinc.com), Mixl‐green fluorescent protein (GFP) HES 11, H9 (WA‐09, WiCell), and iPS (Foreskin)−1 (WiCell) cell lines were cultured as previously described 12. Briefly, human embryonic stem cells (hESCs) and hiPSCs were cultured on mitomycin‐C treated human foreskin fibroblasts (HFF) in knock serum replacer (KSR) media consisting of Dulbecco's modified Eagle's medium (DMEM)/nutrient mixture F‐12, supplemented with β‐mercaptoethanol 0.1 mM, nonessential amino acids 1%, glutamine 2 mM, penicillin 25 U/ml, streptomycin 25 µg/ml, and knockout serum replacement 20% (all from Life technologies, Mulgrave, Australia, www.lifetechnologies.com). All cells were cultured at 37°C 5% CO_2_. Colonies were mechanically dissected every 7 days and transferred to freshly prepared HFFs. Media was changed every second day.

### Neural Induction in Defined Medium

hESCs or hIPSCs were mechanically dissected into pieces approximately 0.5 mm in diameter and transferred to laminin‐coated organ culture plates in N2B27 medium containing 1:1 mix of neurobasal medium with DMEM/F‐12 medium, supplemented with insulin/transferrin/selenium 1%, N2 1%, retinol‐free B27 1%, glucose 0.3%, penicillin 25 U/ml, and streptomycin 25 µg/ml (all from Life Technologies) for 11 days 13. SB431542 (SB; 10 µM, Tocris Bioscience, Bristol, UK, www.tocris.com) was added to the media only for the first 4 days followed by the addition of FGF2 (20 ng/ml, R&D) from days 4 to 11. Cultures were grown on laminin for the first 4 days after which they were dissected into 0.5 mm pieces and cultured in suspension in low‐attachment 96‐well plates (Corning, Corning, NY, USA, www.corning.com) in N2B27 medium.

For CNP induction, neural induction was performed as above and cultures were supplemented with GSK3β inhibitor CHIR99021 (CHIR; 3 µM, Stemgent, Cambridge, MA, USA, www.stemgent.com) from days 0 to 4. For neural crest specification, CNP induction was performed and from days 4 to 11 cultures were supplemented with FGF2 and BMP2 (50 ng/ml; Peprotech, Rehovot, Israel, www.peprotech.com). For floor plate specification, CNP induction was performed and supplemented with the Smoothened agonist (SAG; 400 nM, Merck, Kenilworth, NJ, USA, www.merck.com) from days 0 to 11, from days 4 to 11 cultures were also supplemented with FGF2. For neuronal differentiation of neural crest progenitors cultures were differentiated to neural crest progenitors by day 11, at which time BMP2 was removed and cultures were supplemented with small molecule Rho kinase inhibitor Y27632 (25 µM; Tocris) from days 11 to 18.

For adipocyte, chondrocyte, and osteocyte differentiation, day 11 neural crest progenitors were dissociated with Accutase (Life Technologies) and plated cultured in DMEM 10% FCS medium supplemented with Y27632 (25 µM; Tocris) for 1 week followed by 2 weeks in either: StemPro adipogenesis medium, chondrogenesis medium, or osteogenesis medium (all from Life Technologies).

To determine pathways mediating induction of CNPs, induction was performed in N2B27 medium with SB from days 0 to 4 and instead of CHIR various combinations of the following factors were supplemented: FGF8b (100 ng/ml, R&D Systems, Minneapolis, MN, USA, www.rndsystems.com), IGF1 (20 ng/ml, Peprotech), Wnt3A (100 ng/ml, Peprotech), MEK1/2 inhibitor PD0325901 (PD; 1 µM, Tocris), inhibitor of phosphatidylinositol 3‐kinase (PI3K), and LY294002 (LY; 20 µM, Tocris). Differentiation of these factor conditions toward floor plate or roof plate culture was achieved by removing the factors at day 4 and extending the cultures in the following two ways: For floor plate specification SAG was added to cultures from days 0 to 11 and FGF2 from days 4 to 11. For neural crest specification, BMP2 and FGF2 were added to cultures from days 4 to 11. Where indicated, cultures were supplemented with either: Smoothened agonist (SAG; 400 nM, Merck), FGF8, Wnt3A IFG1 (20 ng/ml, Peprotech), PD (1 µM, Tocris), and LY (20 µM, Tocris). Diagrams of conditions are shown in relevant figures.

### Mesoderm Induction Assay

HES cells were grown on laminin for the first 4 days in N2B27 medium supplemented with CHIR and SB as described above, after which they were cultured in either APEL media (Stemcell Technologies, Tullamarine, Australia, www.stemcell.com) 14 or N2B27 medium, with and without supplementation with FGF2 (20 ng/ml) only, or BMP4 (20 ng/ml)/Activin A (20 ng/ml)/FGF (10 ng/ml), or Activin A (20 ng/ml)/FGF2 (10 ng/ml) for an additional 4–11 days.

### In Ovo Transplantation

Fertilized quail (*Coturnix coturnix japonica*) eggs were obtained from Lago Game Supplies, Vic., Australia. Fertilized chick (*Gallus gallus*) eggs were obtained from Research Hatchery, Vic., Australia. Embryos were staged according to the number of embryonic days (E), Hamburger and Hamilton stages (HH) 15 and, for embryos of E2.5 and younger, by somite counts. Day 11 CNP‐derived neural crest progenitors or CNP‐derived neuroepithelial progenitors were mechanically dissected into small fragments up to the size of an E2 quail somite (200‐µm diameter or less). With use of techniques identical to chick‐quail grafting 16, the fragments were implanted into quail embryos in ovo. For implants next to trunk NT and for vagal level implants E2 (17–20 somite) and E1.5 (7–8 somite) embryos were used, respectively. Electrolytically sharpened 0.25 mm diam. tungsten needles were used to create a slit in which the fragments were inserted.

Gut migration assays were performed by placing CNP‐derived neural crest progenitors at the rostral end of E4.5 (HH25) quail mid/hindguts assembled on 5 × 5 mm^2^ of ethanol‐sterilized black Millipore HA paper. These were inverted tissue‐side down on the chorio‐allantoic membrane (CAM) of 9‐day chick embryos and grown for 7 days as previously described 17. Grafts were then retrieved and dissected free of CAM tissues.

### Temporal Gene Expression Analysis

Total RNA was isolated from individual wells of cells using the RNeasy Micro kit (Qiagen, Chadstone Centre, Australia, www.qiagen.com). Equivalent amounts of total RNA were reverse transcribed using the Quantitect RT kit (Qiagen). Resulting cDNA was then preamplified in multiplex format using gene‐specific primer sets (Supporting Information Methods) prior to qPCR analysis on microfluidic chips. Multiplex pre‐amp reactions consisted of 2.5 µl Taqman Preamp Master Mix (ABI, Life Technologies), 1.25 µl of a 0.2× concentration of pooled Taqman primer sets in TE buffer, and 1.25 µl of cDNA. Preamp cycling conditions were 95°C for 10 minutes, followed by 16 cycles each at 95°C for 15 seconds followed by 60°C for 4 minutes. Reactions were stopped by heating at 99.9°C for 10 minutes. Preamplified samples were diluted 1:5 with Tris‐EDTA (TE) buffer prior to qPCR analysis. Single gene‐specific qPCR reactions using individual Taqman primer sets (Supporting Information Methods) were carried out on a Biomark HD instrument using 48 × 48 Dynamic Array Gene Expression microfluidic chips (Fluidigm, South San Francisco, USA, www.fluidigm.com). Prior to loading onto the chip, individual TaqMan gene expression assays (20×) were diluted 1:1 with 2× assay loading reagent (Fluidigm). Preamplified samples (2.75 µl) were combined with 2.5 µl TaqMan Universal Master Mix (ABI) and 0.25 µl 20× GE sample loading reagent (Fluidigm). qPCR cycling conditions were 50°C for 2 minutes, 95°C for 10 minutes, followed by 40 cycles each at 95°C for 15 seconds followed by 60°C for 1 minute.

### Gene Expression Analysis at Neural Progenitor States

hESC, day 4 and day 11 time points of the various differentiation conditions were collected by mechanical isolation, all conditions were analyzed from three independent biological replicates. Total RNA was isolated from cells using the RNeasy Mini RNA Extraction Kit (Qiagen) and contaminant genomic DNA removed with DNA‐free DNaseI reagents (Ambion, Life Technologies). Primer sequences were designed using the primer3 tool (http://frodo.wi.mit.edu/primer3/). For quantitative reverse transcription and polymerase chain reaction (Q‐PCR), oligo‐dT primed cDNA was synthesized from 130 ng DNaseI‐cleaned total RNA using Murine Moloney Leukemia Virus reverse transcriptase (Promega, Alexandria, Australia, www.promega.com). Q‐RTPCR was performed on an ABI Prism 7500 Fast Real‐Time PCR System (Applied Biosystems, Life Technologies) using Go‐Taq SYBR green master mix (Promega). Relative gene expression values (mRNA fold change) were obtained by normalization to the internal reference genes *RPL32* using the −2^ΔΔCt^ method, where −2ΔΔCt = ΔCt sample − ΔCt calibrator as described 18. Hierarchical clustering and heatmap analysis of Q‐PCR data were done using R‐script and gplots packages.

### Fluorescent‐Activated Cell Sorting Analysis

hESCs or differentiated derivatives were dissociated into single cells with TrypLE Express (Life Technologies) centrifuged and resuspended in 4% paraformaldehyde (PFA) for 10 minutes and subsequently washed in phosphate buffered saline (PBS) and permeabilized with 0.25% Triton X in PBS (PBT). Goat anti‐Sox10 (1:20, R&D Systems) antibody was diluted in blocking solution (PBT with 10% fetal calf serum (FCS)) and cells were centrifuged and resuspended in antibody solution overnight at 4°C. Following three 10‐minute washes in PBT, cells were resuspended in a donkey anti‐goat Cy5 (1:400, Jackson ImmunoResearch, West Grove, PA, USA, www.jacksonimmuno.com) antibodies for 30 minutes at RT, followed by a wash in blocking solution before being sorted using an LSR Fortessa cell analyzer.

### Immunolabeling

Cell monolayers and neurospheres were fixed in 4% PFA for 20 minutes at 4°C and then washed briefly in PBS. Neurospheres were embedded in Tissue‐Tek OCT compound (Labtech, Windsor, Australia, www.labtech.com.au), cut at 10 µm on a cryostat, and sections were placed on superfrost slides. Sections or culture dishes were blocked for 1 hour at room temperature (RT) in blocking solution. The following primary antibodies were used: goat anti‐SOX10 (1:100, R&D Systems), goat anti‐FoxA2 (1:300, Santa Cruz Biotechnology, Dallas, Texas, USA, www.scbt.com), goat anti‐Sox2 (1:500, R&D), mouse anti‐Sox2 (1:500 R&D), mouse anti‐Oct4 (1:100, Santa Cruz), mouse anti‐Tuj1 (1:500, Promega), mouse anti‐Pax3 (1:40, Developmental Studies Hybridoma Bank, Iowa City, Iowa, USA, www.dshb.biology.uiowa.edu), mouse anti‐Pax7 (1:40, DSHB), mouse anti‐AP2 (1:100, DSHB), mouse anti‐Pax6 (1:40, DSHB), mouse anti‐PRPH (1:500, Millipore Merck), mouse anti‐Brn3a (1:500, Millipore), rabbit anti‐Islet1 (1:500, Abcam, Melbourne, Australia, www.abcam.com), rabbit anti‐HOXB1 (1:500 Abcam), mouse anti‐S100β (1:500, Sigma‐Aldrich, Sydney, Australia, www.sigmaaldrich.com), mouse anti‐HuC/D (1:100, Invitrogen/Molecular Probes), mouse anti‐NAPA‐73 (1:200, E/C8, DSHB), rabbit anti‐p75 (1:500, Promega), rabbit anti‐SoxE (1:2,000, Craig Smith, MCRI), goat anti‐BRACHYURY (1:100, R&D Systems), goat anti‐TBX6 (1:100, R&D Systems), and rabbit anti‐Lmx1A (1:5,000, Millipore). Antibodies were diluted in blocking solution incubated on sections overnight at 4°C. Following three 10‐minute washes in PBT, the corresponding Cy5, DyLight‐488, or DyLight‐594 donkey secondary antibodies were applied for 1 hour (overnight for CAM grafts) at RT (1:400, Jackson ImmunoResearch). Sections and cultures were counterstained with 4′,6‐diamidino‐2‐phenylindole (DAPI; 1 µg/ml, Sigma). Slides were mounted in PVA‐DABCO for viewing under a fluorescent microscope (Olympus Life Science, Notting Hill, Australia, www.olympus-lifescience.com), and images captured using the Cell‐M software. Confocal microscopy was performed using an Olympus FV1000 Confocal Microscope. The image was then reconstructed as an intensity projection over the *z*‐axis using Olympus FV10‐ASW 2.0 Viewer software.

### Adipocyte, Chondrocyte, and Osteocyte Staining

Alcian blue, oil red O, and alizarin red S staining were performed according to standard protocols using Alcian blue, 8GX (Sigma), oil red O (Sigma), and Alizarin Red S (Sigma), respectively.

### Statistical Analysis

Fluorescent activated cell sorting (FACS) analysis was performed on at least 10,000 events per replicate. These events were counted after gating out cell debris and doublets on the forward and side scatter. For QPCR and FACS analysis, experiments were repeated at least three times. One‐way ANOVAs or *t* tests were performed for statistical analyses. Quantification of SOX2/BRACHYURY, PAX6, LMX1A, SOX10, or FOXA2‐positive cells was performed on cryostat sections. Cells were stained for their respective markers and the percentage of positive cells was calculated using random sampling of cryostat sections from the aggregates. DAPI nuclei and positive nuclei were counted using image J analysis with Image‐based Tool for Counting Nuclei software.

## Results

### Temporal Gene Expression Changes of SB/CHIR‐Treated hPSCs

**Figure 1:**
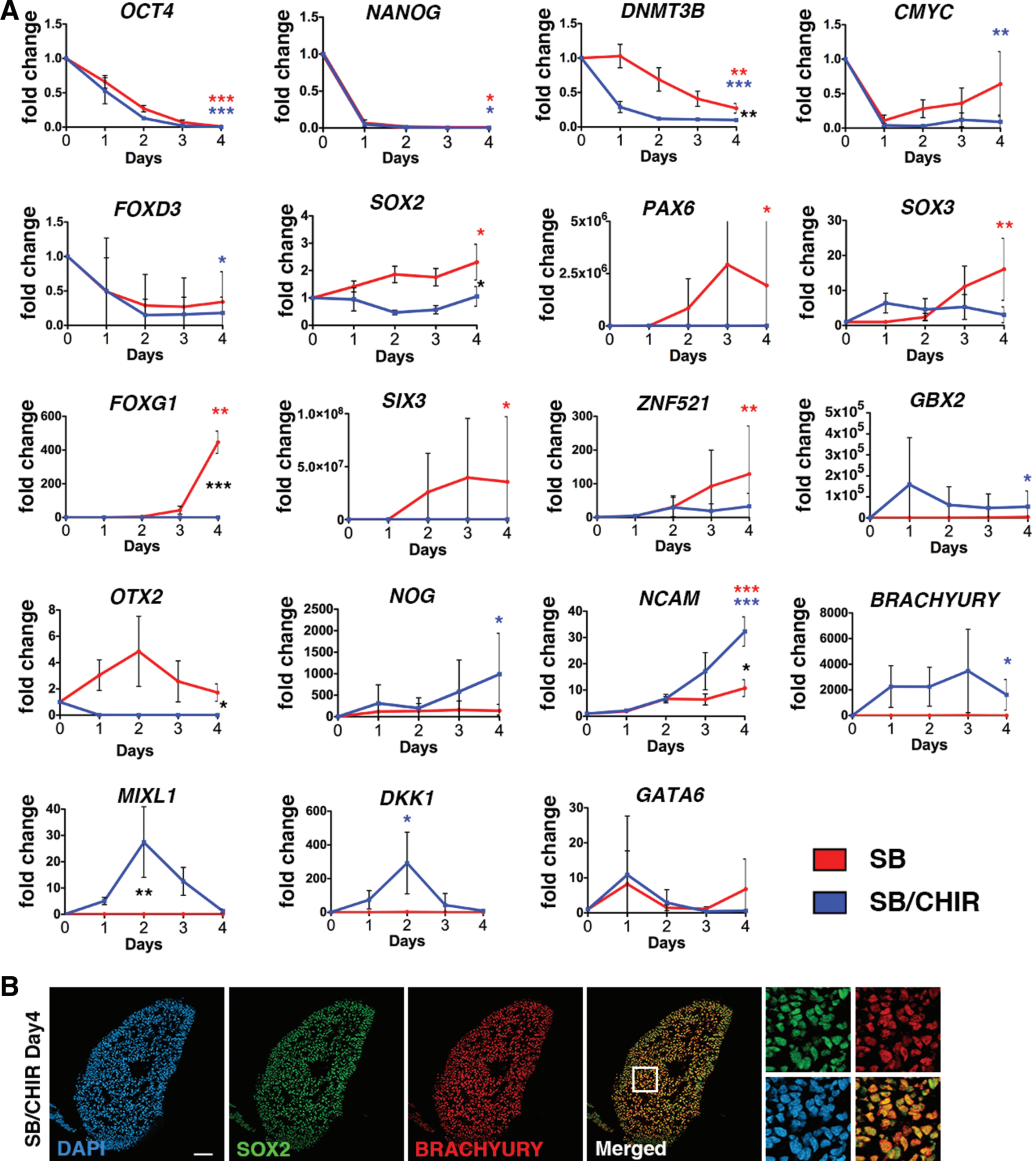
Temporal gene expression analysis comparing SB versus SB/CHIR from pluripotency. QPCR analysis of SB versus SB/CHIR from day 0 to day 4. **(A):** Mean (±SD) ANOVA with Tukey's multiple comparisons were performed. *, *p* <.05; **, *p* <.005; ***, *p* <.0005. Blue stars represent comparison of SB/CHIR to day 0, red stars represent difference between SB to day 0. Black stars represent comparison between SB and SB/CHIR. Not all significant differences are shown. **(B):** Immunocytochemistry analysis of SB/CHIR at day 4 for SOX2 and BRACYURY. Scale bars = 100 µm.

Our previous studies described a novel OCT4−/SOX2+/PAX6− progenitor derived from hPSC, which is induced by dual inhibition of the GSK3β and Activin/Nodal pathways mediated by CHIR and SB treatments, respectively 10. To further characterize the transition from pluripotency to this progenitor state, we performed temporal Q‐PCR analyses of pluripotent and early lineage markers on hPSC treated with SB and CHIR (treatment: SB/CHIR) for 4 days (Fig. 1). This data were compared to hPSC treated with SB only (treatment: SB). We found that within 4 days of SB/CHIR or SB treatments, there was a dramatic and robust decrease in pluripotent gene transcripts, *OCT4* (*p* <.0005), *NANOG* (*p* <.05), and *DNMT3B* (SB *p* <.05, SB/CHIR *p* <.005). Additional pluripotent markers, *C‐MYC* and *FOXD3*, were also significantly decreased in the SB/CHIR group by day 4 (*p* <.005). SOX2 transcript levels remained relatively unchanged in the SB/CHIR group across the 4 days, although they were significantly increased in the SB group relative to both hPSC (day 0) and day 4 SB/CHIR treatments (*p* <.05).

As expected, rostral neuroepithelial markers, *PAX6* (*p* <.05), *SOX3* (*p* <.005), *FOXG1* (*p* <.0005), *SIX3* (*p* <.05), and *ZNF521* (*p* <.005), were all significantly increased in the SB only conditions when compared to the pluripotent stem cell state (Fig. 1). In contrast, expression of rostral neuroepithelial markers was not upregulated in the SB/CHIR conditions, but instead there was a significant increase in the caudal neural marker, *GBX2* (*p* <.05) and persistent expression of *SOX2*. Other genes involved in neural induction, Noggin (*NOG*) (*p* <.05) and *NCAM* (*p* <.05), were also found to be significantly upregulated in the SB/CHIR conditions compared to the pluripotent state. Interestingly, SB/CHIR treatment also showed transient upregulated expression of *MIXL1* at day 2 (*p* <.005) and persistent and massive upregulated expression of *BRACHURY* at day 4 (*p* <.05), both of which are mesoderm lineage markers. Furthermore, SB/CHIR treatment showed coexpression of SOX2/BRACHURY in 97.66% (±0.57 SD) of cells at day 4 (Fig. 1B). The combined expression of caudal neural plate and mesoderm markers in the SB/CHIR conditions suggests the SB/CHIR‐treated hPSCs may be similar to the axial stem cells found in the caudal lateral epiblast region of mouse and avian embryos that have the bipotential to give rise to both neural and mesodermal lineages 4.

### SB/CHIR‐Treated hPSCs Show Biased Differentiation to Caudal Neural Progenitor Lineages

Expression analyses of the SB/CHIR‐treated hPSCs described above showed coexpression of mesoderm and caudal neural progenitor markers. We therefore investigated the potential of SB/CHIR‐treated hPSCs to differentiate to mesodermal progenitor lineages. These experiments were performed using the mesoderm reporter MIXL‐GFP hESC line 11 as well as H9 cells. Following 4 days SB/CHIR treatment, cells were further cultured in media only or media supplemented with BMP4 and Activin A, or Activin A only, to support mesoderm differentiation. Immunostaining analyses of the SB/CHIR aggregates in all differentiation conditions showed no expression of the mesoderm markers, BRACHYURY and TBX6 (data not shown). Although very few GFP+ cells were observed in some of the aggregates, these tended to correspond to apoptotic nuclei (Supporting Information Fig. S1). In contrast, high expression of SOX2+/PAX6+ rosettes was observed (Supporting Information Fig. S1). This suggests that the SB/CHIR‐treated hPSCs are biased toward differentiating to neural lineages despite showing expression of mesodermal markers.

**Figure 2:**
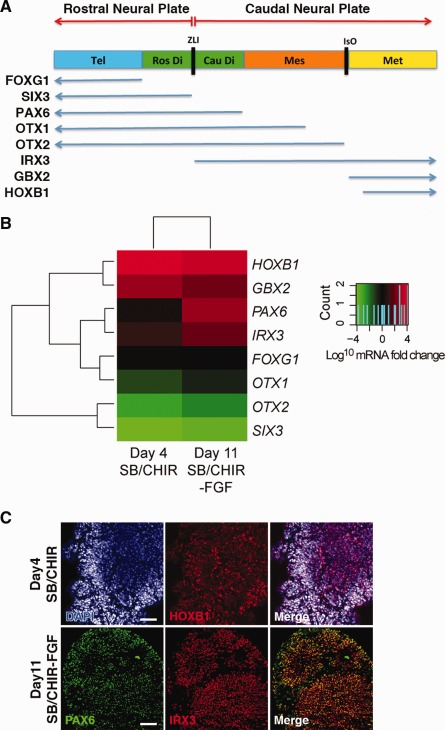
Gene expression analysis of SB/CHIR‐derived neuroepithelial progenitors at day 11. **(A):** Diagram showing gene expression domains along the rostral‐caudal axis of the mouse embryonic neural tube 13, 14, 15, 16, 17, 18, 19, 20, 21, 22. The rostral neural plate gives rise to the Tel and Ros Di, whereas the caudal neural plate gives rise to the Cau Di, Mes, and Met regions of the neural tube. The ZLI region corresponds to the boundary dividing the anterior and caudal neural plate derivatives. **(B):** Two‐way hierarchical clustering of QPCR data for the following genes: *IRX3*, *GBX2*, *HOXB1*, *PAX6*, *FOXG1*, *SIX3*, *OTX2* against treatment conditions: day 4 SB/CHIR and day 11 SB/CHIR‐FGF. The heatmap shows log 10 mRNA fold change values relative to undifferentiated hESCs; upregulated and downregulated genes are colored red and green, respectively. **(C):** Immunocytochemistry analysis of SB/CHIR at day 4 for HOXB1 and SB/CHIR‐FGF at day 11 for PAX6 and IRX3. Scale bars = 100 µm. Abbreviations: Cau Di, caudal diencephalon; Mes, mesencephalon; Met, metencephalon; Ros Di, rostral diencephalon; Tel, telencephalon; ZLI, zona limitans intrathalamica.

We have shown that neural differentiation of SB/CHIR‐treated hPSCs results in upregulated expression of PAX6 10. In the embryo, PAX6 is expressed in both the telencephalon and diencephalon regions of the developing forebrain, which includes the prospective prosomeres 1 and 2 domains that arise from the caudal neural plate 19, 20. We therefore sought to determine whether neural differentiation of SB/CHIR‐treated hPSCs gives rise to neural progenitors that are phenotypic of the rostral or caudal neural plate derivatives. For these analyses, day 4 SB/CHIR‐treated cells were further differentiated for 7 days in FGF‐supplemented neural basal media as previously described 10. Following neural differentiation, Q‐PCR analyses showed significantly high expression of caudal neural tube markers *IRX3*, *GBX2*, *HOXB1* as well as *PAX6* (Fig. 2) 21, 22, 23. By immunofluorescence HOXB1 was detected in SB/CHIR‐treated cells at day4 and following 7 days in FGF, PAX6/IRX3‐positive cells were detected (Fig. 2C). In contrast, no increased expression of rostral neural tube markers, *FOXG1* and *SIX3*, was observed (Fig. 2; Supporting Information Fig. S2) 24, 25, 26. Although *OTX1* and *OTX2* are expressed in the forebrain and midbrain regions of the developing embryo 27, 28, their expression was not significantly changed in the SB/CHIR or SB/CHIR‐FGF‐treated cells (Fig. 2; Supporting Information Fig. S2). This discrepancy may be partly explained by the expression of *OTX2* in undifferentiated hESC 29. Given the caudal neural identity of SB/CHIR‐treated hPSCs and their neural derivatives, we have coined this progenitor population as CNPs.

### Derivation of Neural Crest and Roof Plate Progenitors from CNPs

**Figure 3:**
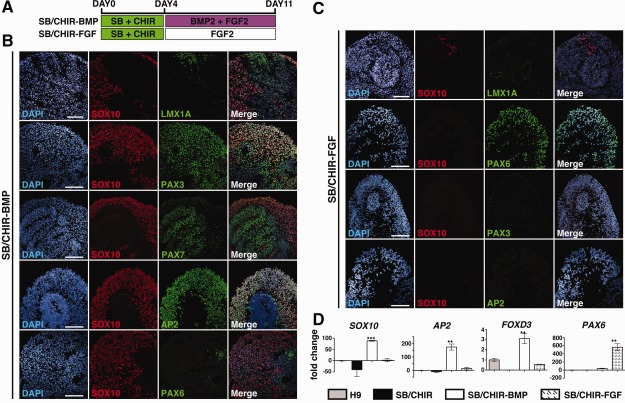
Caudal neural progenitors give rise to neural crest progenitors. **(A):** Diagram of culture conditions. **(B):** Immunocytochemistry analysis of SB/CHIR‐BMP at day 11 for LMX1A, SOX10, PAX3, PAX7, AP2, and PAX6. **(C):** Immunocytochemistry analysis of SB/CHIR‐FGF at day 11 for LMX1A, SOX10, PAX6, PAX3, and AP2. **(D):** QPCR analysis of *SOX10*, *AP2*, *FOXD3*, and *PAX6* at; the pluripotent stage—H9 hESC; day 4 SB/CHIR; day 11 SB/CHIR‐BMP; day 11 SB/CHIR‐FGF. Mean ± SD, *n* = 3, ANOVA with Tukey's multiple comparisons was performed. **, *p* <.005; ***, *p* <.0005. Scale bars = 100 µm.

The embryonic caudal neural plate gives rise to floor plate, roof plate, and neural crest progenitors. We have previously described generation of floor plate cells from CNPs (previously called “pre‐neuroepithelial”) 10. Furthermore, neural differentiation of CNPs gives rise to neuronal progenitors expressing caudal neural tube markers (Fig. 2). Taken together, we hypothesized that CNPs share characteristics to the embryonic neural plate and may therefore also be capable of differentiating to neural crest progenitors given the appropriate signals. To investigate this, we treated CNPs derived from hPSC with either BMP2 or FGF2 for 7 days (treatments SB/CHIR‐BMP and SB/CHIR‐FGF, respectively). After 7 days, aggregate cultures were analyzed for expression of neural crest and neuroepithelial markers by immunostaining (Fig. 3), Q‐PCR (Fig. 3), and FACS (Supporting Information Fig. S3). The BMP‐treated CNPs showed a significantly higher percentage of the early neural crest marker SOX10 by FACS analysis (53.98% ± 7.72% SEM, *p* <.005) compared with FGF‐treated CNPs (5.23% ± 0.65% SEM) (Supporting Information Fig. S2). By Q‐PCR analysis BMP‐treated CNPs also showed significantly higher expression of the early neural crest markers, *SOX10* (*p* <.0001), *AP2* (*p* <.005), and *FOXD3* (*p* <.001), when compared with FGF‐treated CNPs. By immunofluorescence, AP2, PAX3, and PAX7 were detected only in BMP‐treated CNPs (Fig. 3). Of note, PAX3 and PAX7 are markers of the embryonic dorsal neural tube 30, 31. Q‐PCR data show that upregulated expression of neural crest markers occurs after, and not prior to, BMP‐treatment of CNPs (Fig. 3D). In contrast, FGF2‐treated CNPs showed almost no upregulated expression of neural crest and dorsal neural tube markers compared with hESC (Fig. 3D). However, significantly higher expression and percentage of *PAX6* were calculated in the FGF‐treated CNPs (5.29% ± 1.69% SD) compared to the BMP‐treated (36.92% ± 9.44% SD) conditions (*p* <.001; Fig. 3D). Overall, this data demonstrate that exposure of CNPs to BMP proteins specifies their fate to a neural crest lineage at the expense of a PAX6+ neuroepithelial cell lineages.

### Duration of BMP Treatment Is Important for Neural Crest Induction

**Figure 4:**
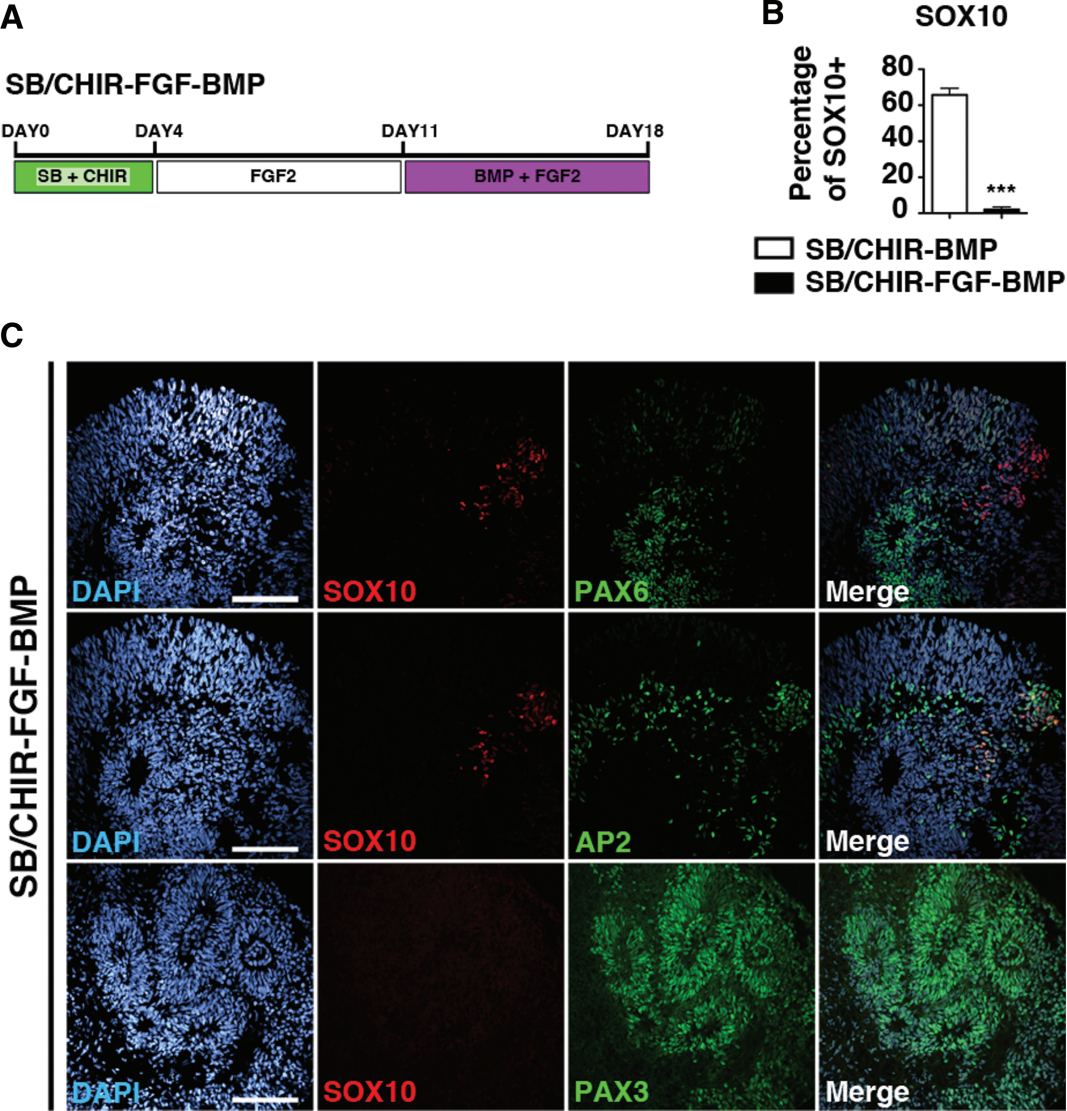
Neural crest specification is restricted to the caudal neural progenitor state. **(A):** Diagram of culture conditions. **(B):** Quantification of SOX10‐positive cell between day 11 SB/CHIR‐BMP condition and day 18 SB/CHIR‐FGF‐BMP condition (mean ± SEM, *n* = 3, *t* test, ***, *p* <.0001). **(C):** Immunocytochemistry analysis of SB/CHIR‐FGF‐BMP at day 18 for, SOX10, PAX6, AP2, and PAX3. Scale bars = 100 µm.

To further determine whether the duration of BMP exposure is important to induce neural crest fate, FGF‐treated CNPs were exposed to BMP2 treatment for a further 7 days in culture (treatment: SB/CHIR‐FGF‐BMP). Interestingly, delayed exposure to BMP2 caused the expression of the dorsal neural tube marker, PAX3 (Fig. 4C), but a significantly lower percentage of SOX10 2.05% (±1.46 SEM; *p* <.0001), compared to day 11 BMP‐treated CNPs (treatment: SB/CHIR‐BMP) (Fig. 4B). This indicates that, similar to the floor plate, hPSC induction of neural crest efficiently occurs prior to their differentiation to PAX6+ neuroepithelial cells.

### Characterization of hPSC‐Derived Neural Crest

**Figure 5:**
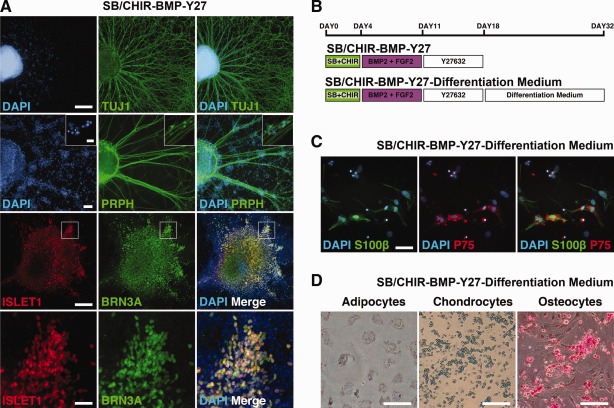
Caudal neural progenitor‐derived neural crest progenitors differentiate into peripheral neurons and Schwann cells. **(A):** Immunocytochemistry analysis of condition SB/CHIR‐BMP‐Y27 at day 18. TUJ1, PRPH, BRN3A, and ISLET1. **(B):** Diagram of culture conditions. **(C):** Immunocytochemistry analysis for Schwann cell, S100β, and P75. **(D):** Differentiation of neural crest progenitors into adipocytes, chondrocytes, and osteocytes, cells stained with oil red O, alcian blue, and alizarin red, respectively (scale bar = 100 µm). Scale bars = Tuj1, BRN3A, and ISLET1 = 500 µm, Peripherin = 100 µm, S100β/P75 = 50 µm, all insets = 20 µm.

**Figure 6:**
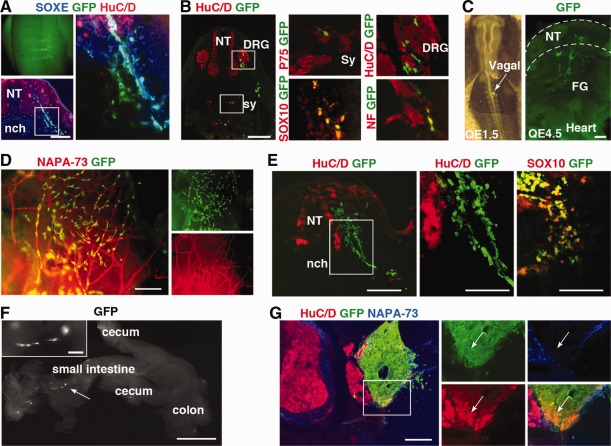
Transplantation of caudal neural progenitor derived neural crest progenitors in ovo and in explant cultures of quail gut tissue. **(A–F):** SB/CHIR‐BMP group and **(G)** SB/CHIR‐FGF group. (A, B, E, G): Transverse section; (C) sagittal slice; (D, F) whole mount. (A): BMP spheres were implanted next to trunk NT at E2 and grown to E5. Cells show extensive hemisegmental migration along endogenous NC pathways. (B): GFP+/SOX10+ cells can be found within endogenous sympathetic chain regions. GFP+/P75+ cells can also be identified and GFP cells identified in DRG and in nerve fiber tracts. (C): BMP spheres were implanted next to NT at vagal level (Somite 3) at E1.5 (9 somite stage) and analyzed at E4.5, showing hemisegmental streams toward foregut. Arrow indicates site of implanted sphere at E1.5. (D): BMP spheres were implanted next to NT at E2 and analyzed at E7, GFP cells migrating with host peripheral axons in flank dermis. (E): BMP spheres were implanted into dorsal NT/NC‐ablated embryos at E2 and analyzed at E5. In the absence of host NC and host DRG, cells from BMP‐treated spheres maintain migration potential. (F): Cells of BMP spheres migrate into gut when placed abutting E4 quail mid/hindgut and grown for 7 days in CAM graft. Arrow indicates a chain of migrating GFP+ cells in the gut wall. (G): Control FGF spheres implanted next to NT do not migrate but forms neural tube‐like structures, with neurons and axons accompanying host axons. Markers: HuC/D = neuron‐specific marker, Sox10, p75 = NC markers, E/C8 = avian‐specific axon marker, NF = neurofilament (axon) marker. Scale bars = (A–E), (G) = 200 µm, (E) and (F) insets = 100 µm, (F) = 1 mm. Abbreviations: DRG, dorsal root ganglion; FG, foregut; GFP, green fluorescent protein; NCC, neural crest cells; nch, notochord; NT, neural tube; Sy, sympathetic chain.

Characteristic functional features of neural crest include their ability to migrate and differentiate to peripheral neurons and non‐neural lineages. Progenitors derived from BMP‐treated CNPs were further differentiated to neurons and glia in vitro (Fig. 5). Neuronal cultures showed extensive outgrowth of Peripherin+ (PRPH) neurites and also high coexpression of sensory neural progenitor markers, BRN3A and ISLET1 (Fig. 5A) 32. Evidence of Schwann cell differentiation was also observed in glial differentiated cultures, with colocalized expression of p75 and S100β (Fig. 5C). BMP‐treated CNPs were also capable of differentiating to mesenchymal lineages, cartilage, adipose, and osteoblasts (Fig. 5D). To assess migration, BMP‐treated CNPs were transplanted beside the embryonic neural tube of avian embryos in ovo. Three to five days post‐transplantation, robust migration of donor cells was observed streaming along endogenous neural crest pathways (Fig. 6A–6F). This migration occurred even in the absence of endogenous neural crest cells (Fig. 6E). Donor GFP+ cells were also localized in regions of peripheral neurons, including the dorsal root ganglia and sympathetic ganglia, and although they were not positive for neuronal markers, they coexpressed SOX10 suggesting they were delayed in their differentiation (Fig. 6B, 6D, 6E). In addition, when BMP‐treated CNPs progenitors were placed abutting E4 quail mid/hindgut and grown for 7 days in CAM graft, donor GFP+ cells migrated into the gut (Fig. 6F). In contrast, in ovo transplants of SB/CHIR‐FGF progenitors remained as tight rosette‐shaped aggregates within the implant (Fig. 6G). Taken together, BMP‐treated CNPs demonstrate in vitro and in vivo properties of bona fide neural crest.

### BMP2‐Treated CNPs Show Properties of Roof Plate Cells

Given that neural crest is derived from the neural folds of the embryo, and these border regions eventually gives rise to the roof plate of the neural tube, we proposed that the BMP‐treated CNPs aggregates consisted of roof plate cells as well as neural crest progenitors. Indeed, 1.45% (±0.45 SD) of cells in the BMP‐treated CNP spheres expressed LMX1A, a marker of roof plate (Fig. 3B). In contrast, significantly fewer LMX1A+ cells were observed in the FGF‐treated conditions (0.17%; ±0.05 SD; *p* <.01; Fig. 3C). To further assess this hypothesis, expression of dorsalizing morphogens was analyzed in the day 4 CNPs, and BMP‐treated and FGF‐treated CNPs. Significantly higher expression of WNT1 and WNT3A was observed in the BMP‐treated CNPs group relative to hPSC (*p* <.005), but not in CNPs (Supporting Information Fig. S4). The FGF‐treated CNPs also showed an increased expression of WNT1 (*p* <.05), although this increase was relatively lower than the BMP treatment (Supporting Information Fig. S4). In contrast, FGF‐treated CNPs showed no significant increase in WNT3A expression. A significant downregulation of BMP 2/4 was observed in both BMP‐CNPs and FGF‐CNPs samples (*p* <.05), although this is cofounded by the fact that BMP2/4 are highly expressed in undifferentiated hPSC (S. Hough, unpublished data). This data suggest that the BMP‐treated CNPs secrete WNTs as dorsalizing signals similar to the embryonic roof plate 33, and thus, similar to the floor plate, these cells are also specified temporally and/or spatially different to PAX6 neuroepithelial cells.

### Pathways Mediating Induction to a CNP State

**Figure 7:**
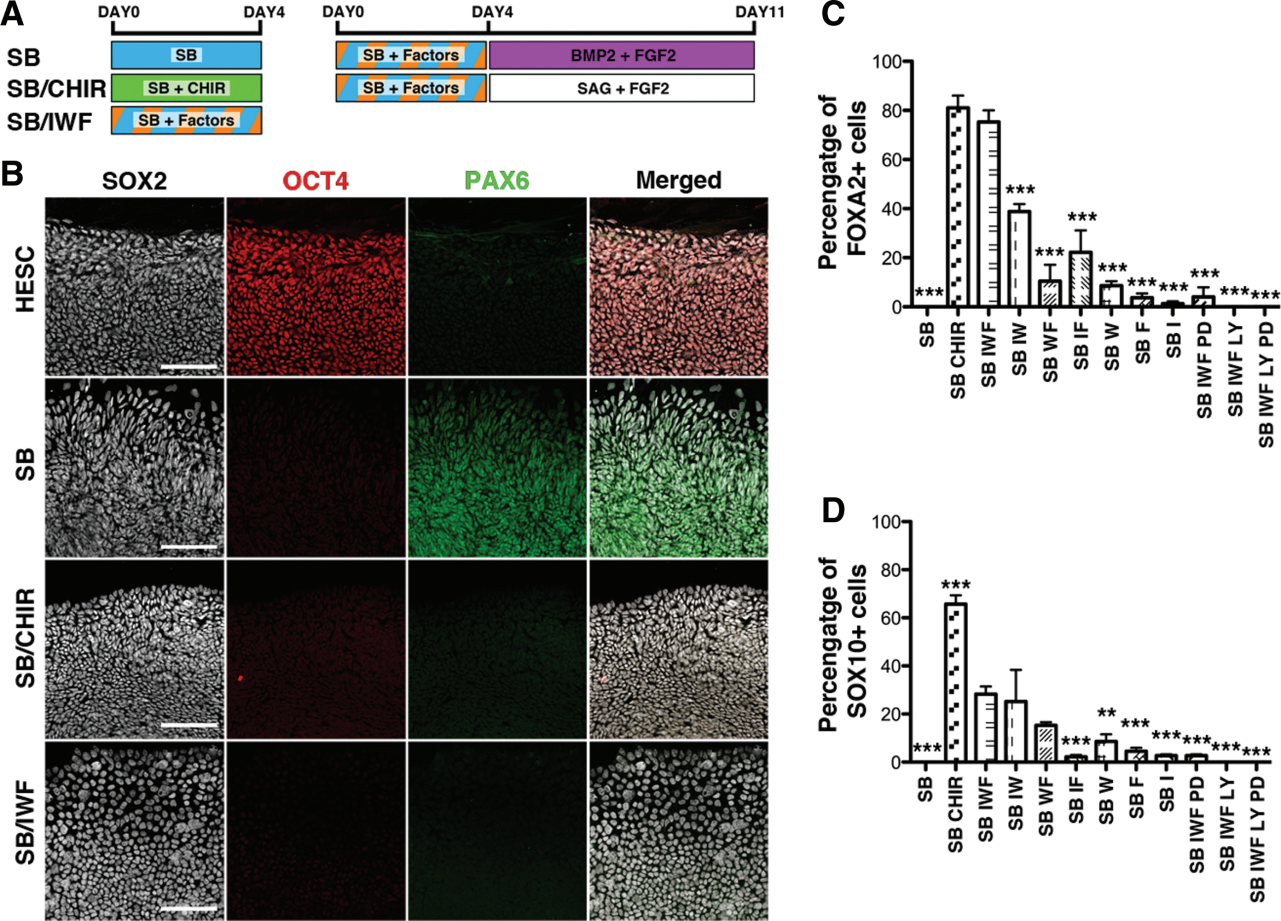
Inducing a caudal neural progenitor state requires IGF1, WNT3A, and FGF8 signaling. **(A):** Diagram of culture conditions. **(B):** Immunocytochemistry analysis for SOX2, OCT4, and PAX6 in conditions; hESC; SB day 4; SB/CHIR day 4; SB/IWF day 4. **(C):** Quantification of FOXA2‐positive cells at day 11. **(D):** Quantification for SOX10‐positive cells at day 11. Mean ± SEM, *n* = 3, one‐way ANOVA with significant differences shown only for comparisons to SB IWF group, **, *p* <.001; ***, *p* <.0001. SB = SB431542, I = IGF1, W = Wnt3A, F = FGF8, LY = LY294002, PD = PD0325901. Scale bars = 100 µm.

Small molecule inhibition of GSK3β may activate many different intracellular pathways including, canonical Wnt, and IGF signaling 34. To identify the specific pathways involved in the neural induction of CNPs, we examined whether extrinsic factors known to be involved in embryonic neural induction could mimic or replace GSK3β inhibition by CHIR. hPSCs were treated with the small molecule inhibitor SB together with different combinations of Wnt3a, FGF8, and IGF for 4 days. Each of these conditions was then exposed to either SAG (a small molecule agonist of the SHH pathway) or BMP2 for a further 7 days, to induce their fate toward floor plate or neural crest, respectively. Proportion of cells expressing SOX2+/OCT4−/PAX6− was observed to be the highest at day 4 when all three factors (Wnt3a, FGF8, and IGF) were present (Fig. 7B). Furthermore, these conditions gave rise to significantly higher FOXA2 expression when treated with SAG (Fig. 7C) and significantly higher SOX10 expression when treated with BMP2 (Fig. 7D). Omission of FGF8 showed similar results, although floor plate induction was reduced. Floor plate or neural crest induction was significantly reduced or abolished if IGF1 and/or Wnt3a were omitted. These results were further supported using small molecule inhibitors of the PI3K pathway (LY294002) and MEK1/2 pathway (PD0325901), which block IGF1 and FGF signaling, respectively 35, 36, 37. Taken together, these results show that hPSC induction to CNPs is mediated by the canonical Wnt, IGF1, and FGF8 pathways.

## Discussion

These studies describe a novel progenitor type derived from hPSC that shows many similarities to the embryonic caudal neural plate. These CNPs can be derived directly from a pluripotent state through the small molecule inhibition of GSK3β and Activin/Nodal signaling. CNP progenitors are a pivotal point for determining cell fate to roof plate/neural crest, floor plate, and caudal neuroepithelial progenitors depending on the morphogenetic signal. The key gene expression profile that defines CNPs is the expression of SOX2 and BRACHURY and absence of pluripotent associated genes and PAX6. Furthermore, we determined that the induction of CNPs from hPSC involves the combination of canonical Wnt, IGF1, and FGF8 pathways.

Our study has identified the mechanisms by which CHIR is able to mediate induction of CNPs from hPSC. Previous studies have suggested that CHIR activates canonical Wnt signaling, which caudalizes hPSC‐derived neural progenitors 9, 38. This hypothesis is based on the observation that hPSC neural induction via SMAD inhibition only generates forebrain neural progenitors and thus, the caudal shift mediated by CHIR treatment corresponds to spatial positions of neural crest and floor plate cells along the embryonic A‐P neural tube axis. Consistent with this theory, our expression data of day 4 CHIR‐treated hESC, that is CNPs, do show a significant increase in the hindbrain marker, *GBX2*, as well as the mesoderm progenitor marker, *BRACHYURY*. This expression profile may be somewhat similar to the bipotential axial stem cells found in the caudal regions of amniote (avian and mouse) embryos 4. Both Sox2 and Brachyury are expressed in these cells, which are located in the regions adjacent to the primitive streak within the caudal neural plate 5. It is proposed that Wnt3a signaling combined with BMP repression via NOG released from the node induces Sox2 expression in the axial stem cells to drive neural fate. Whereas, axial cells migrating through the primitive streak are less exposed to Wnt signals, and this mediates both the downregulation of Sox2 and a corresponding increase in Tbx6 expression, thereby inducing mesoderm fate 4. In our study, the CNPs show a bias to differentiate toward neural lineages rather than mesoderm. The disparity in differentiation potential between embryonic axial stem cells and hPSC‐derived CNPs may be due to differences in the levels of canonical Wnt signaling and/or the activation of other signaling pathways.

In our studies, the signaling pathways required to derive CNPs from hPSC were determined. We found that CHIR treatment of hPSC could be replaced by combined treatment with extrinsic factors Wnt3a, IGF1, and FGF8. Indeed, canonical Wnt signaling inhibits GSK3β 39. Similarly, FGF8 and IGF1 also inhibit GSK3β via Mek/Erk‐p90RSK and PI3K/Akt pathways, respectively 40, 41, 42. The PI3K/Akt pathway also represses SMAD2/3 signaling 37. The involvement of these signaling pathways in deriving CNPs was supported by our findings showing that small molecule inhibitors of Erk/Mek and PI3K/Akt repressed induction of CNPs and subsequently the neural crest and floor plate derivatives. Furthermore, previous reports have shown that Gsk3β inhibition together with Activin signaling promotes hPSC differentiation toward mesoderm via the canonical WNT and SMAD2/3 pathways 37. Since SB is an inhibitor of SMAD2/3 signaling, this blocks hPSC differentiation to mesoderm. SMAD2/3 inhibition also induces expression of rostral neuroepithelial markers; however, this is prevented in our system because of the simultaneous inhibition of GSK3β signaling. Therefore, in essence, dual inhibition of GSK3β and SMAD2/3 pathways induces hPSC to differentiate directly to CNPs, whilst transiently repressing lineage determination toward mesoderm and neural fates. It is during this early caudal state prior to PAX6 expression that exposure to morphogens, such as BMP and SHH, will direct CNP fate to neural crest/roof plate or floor plate lineages, respectively. Whereas, once CNPs have differentiated to PAX6+ caudal neuroepithelial cells, activation of BMP and SHH signaling will induce their differentiation to dorsal and ventral neural progenitors, respectively.

Consistent with our findings, previous studies also showed that neural crest induction occurred prior to and at the expense of rosette‐forming CNS neuroepithelial cells 43, 44. Studer and colleagues described a method for inducing neural crest and melanocyte precursors from hPSC using combined inhibition of BMP and TGFβ signaling for 2–3 days followed by CHIR treatment for another 8 days 43. Specification of neural crest to melanocytes was induced by exposure of BMP4 and endothelin3 during CHIR treatment. Dalton and colleagues also described a similar protocol for generating neural crest, which involved activation of the canonical Wnt pathway and SMAD inhibition 44. Although the methodologies are very similar, our study identified a specific progenitor stage during hPSCs neural crest induction, which is the same progenitor type for induction to floor plate and neuroepithelial lineages. What determines CNP fate to dorsal, ventral, or intermediate cell types depends on the subsequent extrinsic signaling factors.

## Conclusions

In summary, this study has identified and defined an early CNP state that mimics the embryonic caudal neural plate region and is pivotal for cell fate toward major progenitor lineages of the central and peripheral nervous system. Overall, these studies reveal the key players and pathways that underpin neurogenesis and stem cell fate.

## Author Contributions

M.denham and M.Dottori: concept and design, financial support, collection and/or assembly of data, data analysis and interpretation, manuscript writing, and final approval of manuscript; K.H.: collection and/or assembly of data, data analysis and interpretation, and final approval of manuscript; T.M., B.R., D.Z., S.H., S.I., A.A., F.F., J.L., and D.E.: collection and/or assembly of data and final approval of manuscript; D.F.N.: financial support, data analysis and interpretation, and final approval of manuscript; M.F.P.: financial support, data analysis and interpretation, and final approval of manuscript.

## Disclosure of Potential Conflicts of Interest

The authors indicate no potential conflicts of interest.

## Supporting information

Supplementary Information Figure 1

Supplementary Information Figure 2

Supplementary Information Figure 3

Supplementary Information Figure 4

Supplementary Information Legends

Supplementary Information Methods
