# Supplementary figures and images for: Multipotent Caudal Neural Progenitors Derived from Human Pluripotent Stem Cells That Give Rise to Lineages of the Central and Peripheral Nervous System
Source: Stem Cells. 2015 May 21;33(6):1759–70. doi: 10.1002/stem.1991 (PMC5347855; doi:10.1002/stem.1991)

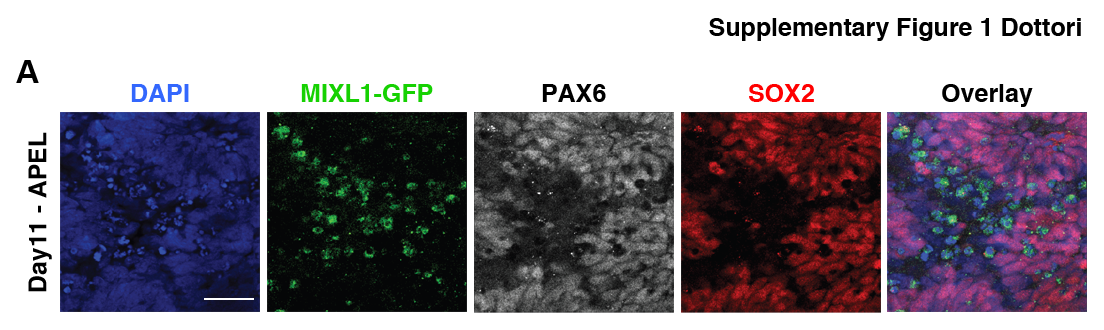

Supplement: Supplementary file 1 — Supplementary Information Figure 1 [file STEM-33-1759-s001.tif]

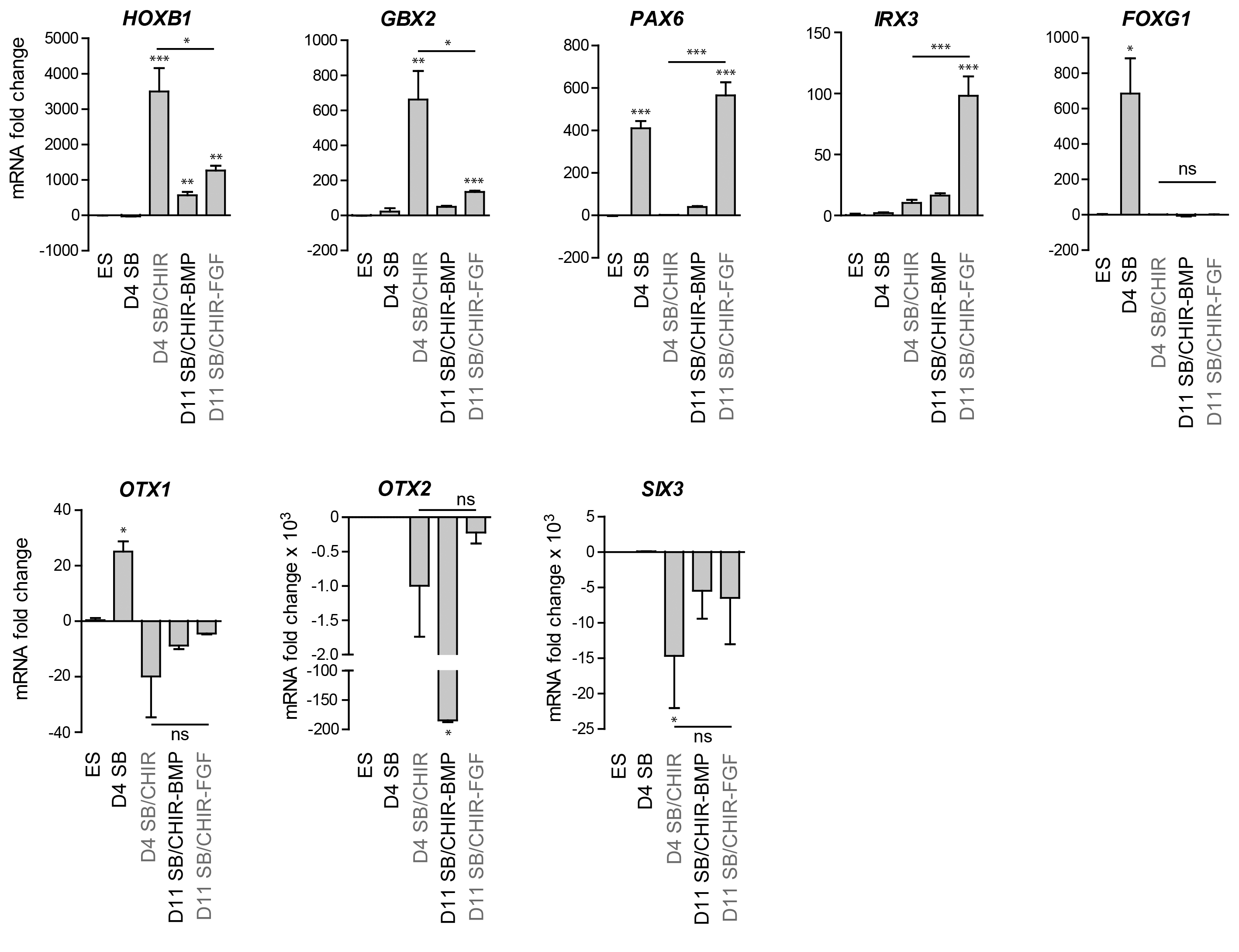

Supplement: Supplementary file 2 — Supplementary Information Figure 2 [file STEM-33-1759-s002.tif]

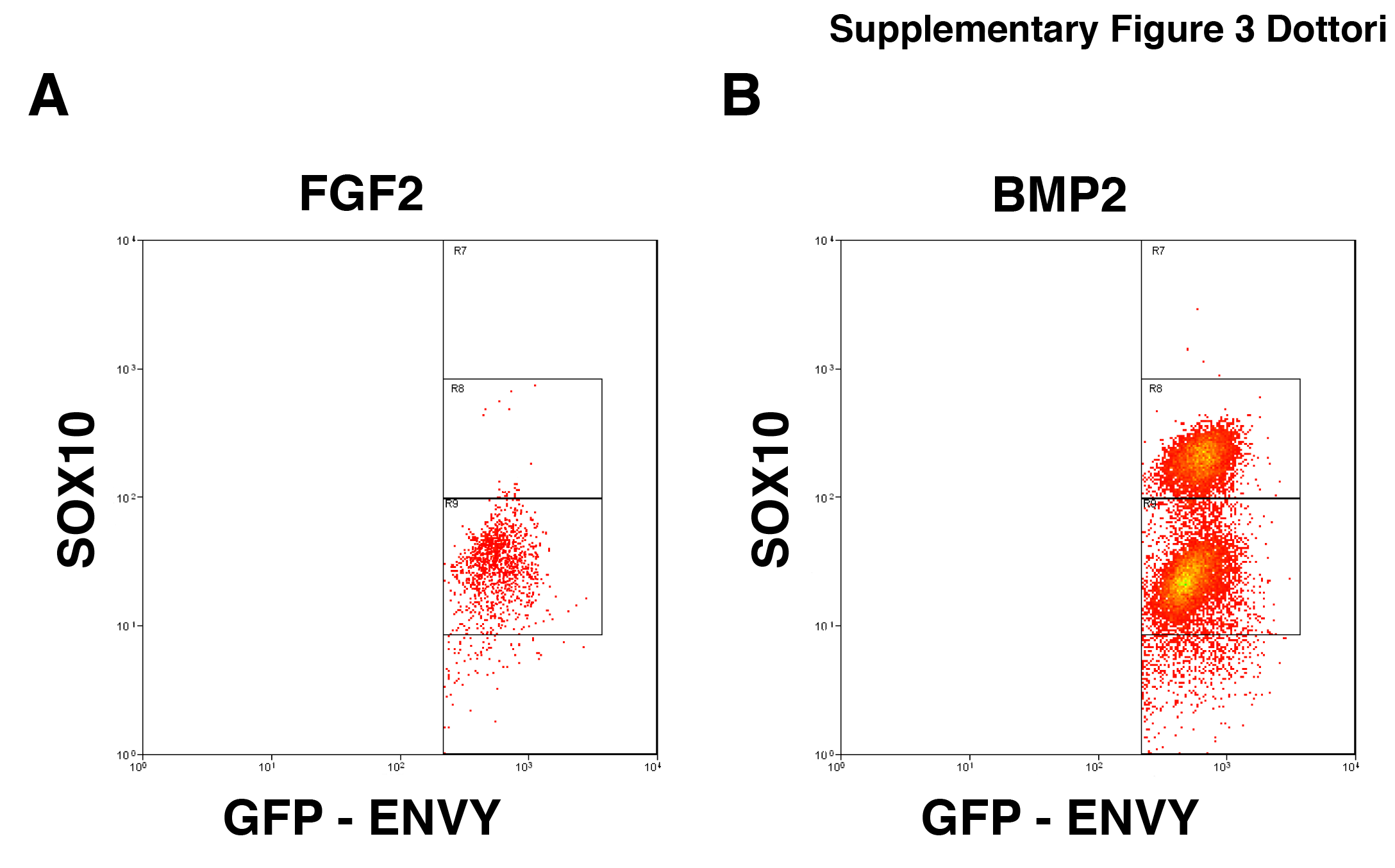

Supplement: Supplementary file 3 — Supplementary Information Figure 3 [file STEM-33-1759-s003.tif]

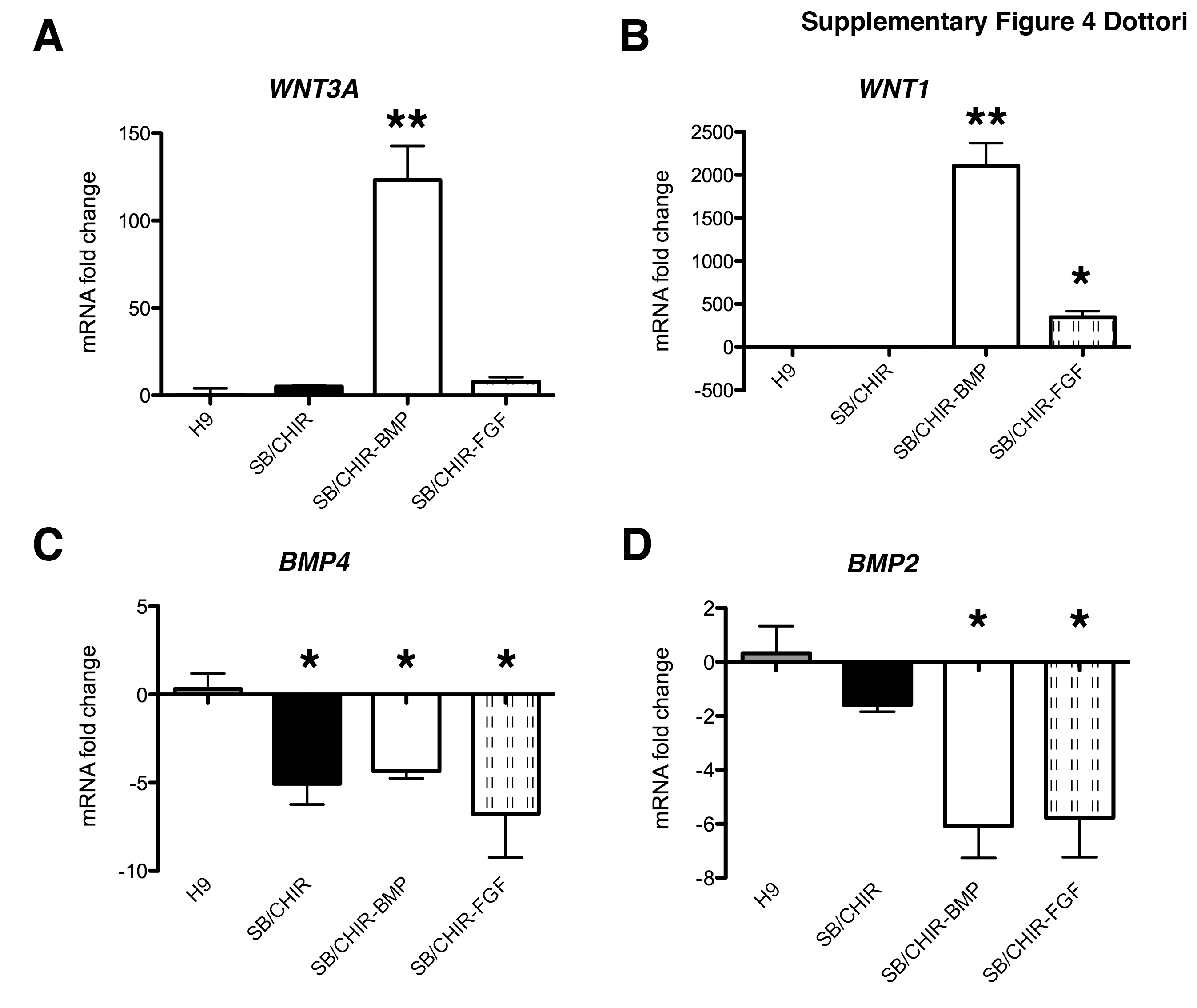

Supplement: Supplementary file 4 — Supplementary Information Figure 4 [file STEM-33-1759-s004.tif]
