# Supplementary material for: Multipotent Caudal Neural Progenitors Derived from Human Pluripotent Stem Cells That Give Rise to Lineages of the Central and Peripheral Nervous System
Source: Stem Cells. 2015 May 21;33(6):1759–70. doi: 10.1002/stem.1991 (PMC5347855; doi:10.1002/stem.1991)
Supplement: Supplementary file 5 — Supplementary Information Legends [file STEM-33-1759-s005.doc]

**Supplementary Figure Legends**

**Supplementary Figure 1:**

**Assessment of CNPs mesodermal competence.**

Using MIXL-GFP HES cells, Day 4 SB/CHIR CNPs were cultured in APEL medium until day 11 and assessed by immunofluorescence for MIXL, SOX2 and PAX6 expression. PAX6/SOX2 positive cells were mutually exclusive to GFP. Apoptotic nuclei co-localise with GFP. Scale bar: 50um

**Supplementary Figure 2:**

**QPCR analysis of CNPs and their day 11 derivatives.**

Undifferentiated hESCs, SB only condition at day4, SB/CHIR condition at day4, SB/CHIR-BMP condition at day11 and SB/CHIR-FGF condition at day 11 were analysed by QPCR for *HOXB1*, *GBX2*, *PAX6*, *IRX3*, *FOXG1*, *OTX1*, *OTX2* and *SIX3* transcripts. Histograms show mean mRNA fold change relative to undifferentiated hESCs after normalisation to the internal reference gene, *RPL32*. Error bars (± S.E.M); asterisks where present indicate statistical significance, * P<0.05, ** P<0.01, *** P<0.001 (t-test).

**Supplementary Figure 3:**

**Quantification of cell positive for SOX10 by FACS analysis**

Using the GFP ENVY cell line, spheres at day 11 from the SB/CHIR-BMP and SB/CHIR-FGF treatment conditions were sorted for GFP and SOX10 after immunolabelling for anti-SOX10. **a)** SB/CHIR-FGF spheres were 5.23% (±0.65 SEM) positive for SOX10. **b)** SB/CHIR-BMP spheres were 53.98% (±7.72 SEM) positive for SOX10, significantly greater than SB/CHIR-FGF group (P<0.005).

**Supplementary Figure 4:**

**QPCR analysis of Neural Crest Progenitors.**

Undifferentiated H9 HES cells, SB/CHIR condition at day4, SB/CHIR-BMP condition at day11 and SB/CHIR-FGF condition at day 11 were analysed by QPCR for *WNT3A*, *WNT1*, *BMP4* and *BMP2* transcripts. **a)** *WNT3A*, SB/CHIR-BMP treatment at day11 showed a significantly higher fold change compared to HESCs. **b)** *WNT1*, SB/CHIR-BMP treatment and SB/CHIR-FGF at day11 showed a significantly higher fold change compared to HESCs. **c)** *BMP4*, SB/CHIR at day4, SB,CHIR-BMP and SB/CHIR-FGF at day11 showed a significantly lower fold change compared to HESCs. **d)** *BMP2*, SB/CHIR-BMP and SB/CHIR-FGF at day 11 showed a significantly lower fold change level compared to HESCs. (means ± S.E.M, t-test for significant levels (* P<0.05, ** P<0.005)
