# Supplementary material for: Multipotent Caudal Neural Progenitors Derived from Human Pluripotent Stem Cells That Give Rise to Lineages of the Central and Peripheral Nervous System
Source: Stem Cells. 2015 May 21;33(6):1759–70. doi: 10.1002/stem.1991 (PMC5347855; doi:10.1002/stem.1991)
Supplement: Supplementary file 6 — Supplementary Information Methods [file STEM-33-1759-s006.docx]

**Supplementary Methods**

| **TaqMan primer sets for multiplex Fluidigm Q-PCR analysis** | | |  |
| --- | --- | --- | --- |
|  |  |  |  |
|  | **GENE** | **Name** | **ABI Assay ID** |
| 1 | 18S | Eukaryotic 18S rRNA | Hs99999901_s1 |
| 2 | DKK1 | dickkopf homolog 1 (Xenopus laevis) | Hs00183740_m1 |
| 3 | DNMT3b | DNA (cytosine-5-)-methyltransferase 3 beta | Hs00171876_m1 |
| 4 | FOXD3 | forkhead box D3 | Hs01027393_s1 |
| 5 | FOXG1 | forkhead box G1 | Hs01850784_s1 |
| 6 | GAPDH | Human GAPD (GAPDH) Endogenous Control (FAM / MGB Probe, Non-Primer Limited) | Hs99999905_m1 |
| 7 | GATA4 | GATA binding protein 4 | Hs00171403_m1 |
| 8 | GATA6 | GATA binding protein 6 | Hs00232018_m1 |
| 9 | GBX2 | gastrulation brain homeobox 2 | Hs00230965_m1 |
| 10 | MIXL1 | Mix1 homeobox-like 1 | Hs00430824_g1 |
| 11 | MYC | v-myc myelocytomatosis viral oncogene homolog (avian) | Hs00153408_m1 |
| 12 | NANOG | Nanog homeobox | Hs02387400_g1 |
| 13 | NCAM | neural cell adhesion molecule 1 | Hs00941821_m1 |
| 14 | Noggin | noggin | Hs00271352_s1 |
| 15 | OCT3/4 | POU class 5 homeobox 1 | Hs00999632_g1 |
| 16 | OTX2 | orthodenticle homeobox 2 | Hs00222238_m1 |
| 17 | PAX6 | paired box 6 | Hs00240871_m1 |
| 18 | PPIA | peptidylprolyl isomerase A (cyclophilin A) | Hs99999904_m1 |
| 19 | SIX3 | SIX homeobox 3 | Hs00193667_m1 |
| 20 | SOX1 | SRY (sex determining region Y)-box 1 | Hs01057642_s1 |
| 21 | SOX2 | SRY (sex determining region Y)-box 2 | Hs01053049_s1 |
| 22 | SOX3 | SRY (sex determining region Y)-box 3 | Hs00271627_s1 |
| 23 | T | T, brachyury | Hs00610080_m1 |
| 24 | ZNF521 | zinc finger protein 521 | Hs00296682_m1 |

**QPCR primer sequences**

| **Gene** | **Sense** | **Antisense** | **Product Size** |
| --- | --- | --- | --- |
| SOX10 | GCTGCTGAACGAAAGTGACAAG | TCTTGTAGTGGGCCTGGATG | 197 |
| AP2 | ACTCGGAGACCTCTCGATCC | GGACACGGGGCCTTTCTTAAT | 115 |
| FOXD3 | CCCAAGAACAGCCTAGTGAAGC | TTCTCCCTGTAGTAGGGGAAGC | 140 |
| PAX6 | GGTGAATGGGCGGAGTTATGA | CAGGGGAAATGAGTCCTGTTGA | 110 |
| WNT3A | CATCAAGATTGGCATCCAGGAG | ACTCCCTGGTAGCTTTGTCCAG | 114 |
| WNT1 | CTTCGGCAAGATCGTCAACC | GATTCGATGGAACCTTCTGAGC | 114 |
| BMP4 | ATCTTTACCGGCTTCAGTCTGG | AGATGTTCTTCGTGGTGGAAGC | 124 |
| BMP2 | AGAAACGAGTGGGAAAACAACC | AAGCATCTTGCATCTGTTCTCG | 116 |

SIX3 AGTGGTACCTACAGGACCCCTACC GTGCTGGAGCCTGTTCTTGG 149

FOXG1 AAGAACGGCAAGTACGAGAAGC GCTTGTTCTCGCGGTAGTAAGG 145

GBX2 CAAAGGCTTCCTGGCCAAAG CCTTTGACTCGTCTTTCCCTTG 110

IRX3 ACAGATCGCTGTAGTGCCTTGG TGAGGAGAGAGCCGATAAGACC 120
